# Pre-treated theaflavin-3,3′-digallate has a higher inhibitory effect on the HCT116 cell line

**DOI:** 10.1080/16546628.2017.1400340

**Published:** 2017-11-15

**Authors:** Yangping Ding, Bingcan Chen, Zili Gao, Huayi Suo, Hang Xiao

**Affiliations:** ^a^ College of Food Science, Southwest University, Chongqing, China; ^b^ Department of Food Science, University of Massachusetts, Amherst, MA, USA; ^c^ Department of Plant Sciences, North Dakota State University, Fargo, ND, USA

**Keywords:** theaflavin-33′-digallate, HCT116, cell cycle, apoptosis, Western blot

## Abstract

The pro-apoptotic and inhibitory effects of the aflavin-3,3′-digallate (TFDG), which is the typical pigment in black tea, have been demonstrated in many cancer cell lines. However, TFDG is not stable in general culture conditions. So, to what extent TFDG or which degradation products of TFDG play an antitumor role is still unclear. In this study, we evaluated the effect of different treatments of TFDG on HCT116 cells. Compared with the control, both TFDG and O-TFDG (the TFDG that was pre-incubated in an incubator at 37°C for 3 hbefore adding into 96-well plates) significantly inhibited HCT116 cell growth. However, pre-treated TFDG was far better than TFDG. The IC50 values of TFDG and O-TFDG-3 were 17.26 μM and 8.98 μM, respectively (the cells were treated by O-TFDG for only 3 h, after which the media were replaced by fresh media for another 69 h incubation). Cell-cycle analysis revealed that 20 μM of O-TFDG and O-TFDG-3 caused cell-cycle arrest at G2 phase in HCT116 cells. Western blot analysis also demonstrated that the anti-inflammatory effect of O-TFDG-3 is stronger than that of TFDG by decreasing COX-2 and iNOS. On the other hand, O-TFDG induced HCT116 cells apoptosis mainly by increasing the expression of p53, p21, and cleaved caspase-3. The current study demonstrated that O-TFDG had a higher inhibitory effect on HCT116 cells than TFDG, and sowe may inferfromthis that the degradation products of TFDG play a key role against tumors.

Colorectal cancer (CRC) is one of the leading causes of cancer deaths worldwide [1]. Although surgical resection is still the best choice for treatment of CRC, subsequent chemotherapy is required to relieve symptoms and prolong life [2]. Recently, a number of epidemiological and animal model studies have demonstrated that polyphenols from fruits and vegetables are strikingly important in reducing the risk of CRC. Theaflavins (TFs) are characteristic compositions from black tea and are responsible for its color, astringency, and taste [3]. The typical pigments, mainly including theaflavin (TF), theaflavin-3-gallate (TF-3-G), theaflavin-3′-gallate (TF-3′-G), and theaflavin-3,3′-digallate (TFDG), are formed by oxidative condensation between catechol-type catechins and pyrogallol-type catechins during fermentation. Among theaflavins, TFDG is generally regarded as the most effective component for the inhibitory effects against carcinogenesis without adverse side effects by affecting multiple signal transduction pathways, such as upregulating p53 and p21, inhibiting phosphorylation of the cell survival protein Akt and MAPK pathway, downregulation of NF-kappa B, shifting the ratio between pro-/antiapoptotic proteins, and so on [4–6].

It has been documented that p53 is mutated in approximately 50% of all human tumors [7], and loss of normal p53 function is involved in heredity [8]. TFs could induce apoptosis of p53 mutated breast cancer cells by activing the Fas death receptor/caspase-8 pathway and inhibiting the pAkt/pBad cell survival pathway [9]. TFs could also inhibit human breast cancer cell migration by activating the p53/ROS/p38MAPK positive feedback loop that hinders NF-κB/p65 via the p53-ROS feedback loop to finally inhibit pro-migratory enzymes MMP-2 and MMP-9 [10]. Phospho-ERK1/2 and phospho-MEK1/2 are pivotal signaling proteins in the Ras-activated signal transduction pathway. TFDG could cause a rapid and sustained decrease in phospho-ERK1/2 and -MEK1/2 protein expression. The inhibitory effect of TFDG was more rapid than (–)-epigallocatechin-3-gallate (EGCG). TFDG could potently decrease levels of Raf-1 protein, which is a serine/threonine protein kinase directly upstream of MEK1/2 [11,12]. In addition, Raf-1 is a protein complex consisting of heat-shock protein 90 (hsp90) and p50 [13]. TFDG might promote degradation of the Raf-1-hsp90 complex. Finally, these mechanisms lead to a decrease in phospho-MEK1 and phospho-ERK1/2 levels, and inhibit the signal transduction to the nucleus [12].

TFs could also play a role against tumors by inhibiting the reactivity of related enzymes. 5α-reductase can convert testosterone into dihydrotestoterone (DHT), which subsequently binds to the androgen receptor (AR) (a ligand-dependent transcriptional factor), contributing to prostate cancer development and progression [14]. TFDG could inhibit 5α-reductase by interacting with the NADPH binding site [15]. The aromatase plays an important role in the growth of hormone-dependent breast carcinoma by transforming androstenedione into estrone. Theaflavins significantly inhibit aromatase activities of human placental and rat ovarian tissue. In addition, theaflavins decrease antioxidant MnSOD and increase pro-oxidant proline oxidase in Wt-p53 MCF-7 cells, but the same change has not been observed in mutant p53-containing MDAMB-231 cells and p53-knock-out MCF-7 cells. This clearly suggests that theaflavins maintain the balance between pro- and antioxidant enzymes in a p53-dependent manner to generate ROS in MCF-7 cells [10].

Although TFDG is a good antitumor agent, few researchers have considered the instability of TFDG in the course of antineoplastic experiments to date. Due to the molecular nature of TFDG, it degrades rapidly within a short time under general experimental conditions [16]. Nevertheless, the duration of almost all the experiments using TFDG against tumors *in vitro* and *in vivo* was over 24 hours. Thus, the contribution of parent TFDG and/or its degradation products toward its anticancer effects have not been quantified until now. In the present study, we studied the effect of different treatments of TFDG on HCT116 cell line.

## Materials and methods

### Cell lines and treatments

Theaflavins (purity ≥20%) were purchased from Hangzhou Easily Biotechnology Co. Ltd (Hangzhou, China). Human colon cancer cells HCT116 were obtained from American Type Cell Collection (ATCC, Manassas, VA). HCT116 cells were maintained in RPMI 1640 media (ATCC) supplemented with 5% fetal bovine serum (Mediatech, Herndon, VA), 100 U/ml of penicillin, and 100 μg/ml streptomycin (Sigma-Aldrich). The cells were cultured in a 5% CO_2_–95% air humidified atmosphere at 37°C. Antibodies for cyclin D1, cyclin E, COX-2, iNOS, cleaved caspase-3, and β-actin were obtained from Santa Cruz Biotechnology (Santa Cruz Biotechnology, CA); p21, p53 primary antibodies were purchased from Cell Signaling Technology (Beverly, MA). All chemicals were purchased from Fisher Scientific, unless otherwise specified. HCT116 cells were used within 3–30 passages.

### Preparation of TFDG

**Figure 1.**
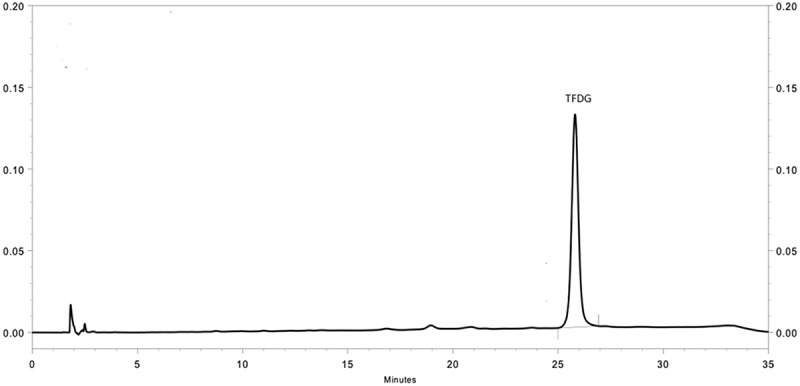
HPLC chromatogram of TFDG.

Preparation of TFDG was carried out according to our previous study with slight modifications. Briefly, theaflavins (5 g) were subjected to resin NKA-9 column chromatography (60 × 3 cm) with 30% ethanol and 60% ethanol, respectively. The 60% ethanol elution was concentrated (1 g) and further purified by polyamide (80–120 mesh) column chromatography (40 × 2 cm) with chloroform:acetone:methanol (5:5:3) to yield TFDG (100 mg) as a yellow amorphous powder. The HPLC chromatogram of TFDG is shown in Figure 1, and the purity of TFDG was found to be over 95%.

### Stability of TFDG in the media

To assess the stability of TFDG in cell-culture conditions, TFDG was added to 50 ml of RPMI media at a final concentration of 50 μM in triplicate and incubated at 37°C. A 0.5 ml sample was taken every 30 min for 3 h. Samples were extracted immediately with an equal volume of ethyl acetate, and this was repeated three times. Ethyl acetate layer was collected, and the solvent was evaporated through a rotary vacuum evaporator. The sample was then dissolved using 0.5 ml of methanol. All sample solutions were filtered through a 0.45 μm filter for HPLC analysis. All chromatographic analyses were carried out on an HPLC-UVD system consisting of a C18 column (5 μm, 150 mm × 4.6 mm diameter). The column temperature was set at 40°C. Binary gradient elution was carried out using mobile phases consisting of 2% (v/v) acetic acid in water (Solvent A) and acetonitrile/ethyl acetate (ratio 7:1 v/v) (Solvent B). The flow rate was maintained at 1 ml/min. Separations were performed for 35 min using the following linear gradient: 82% A at 0 min, 70% A at 26 min, and 82% A from 30 to 35 min. The injection volume and UV detection wavelength were 10 μl and 280 nm, respectively.

### Cell-viability analysis

Cell viability was assessed using a variation of the 3-(4,5-dimethylthiazol-2-yl)-2,5-diphenytetrazolium bromide (MTT, Sigma-Aldrich) assay. HCT116 cells were seeded into 96-well plates (2 × 10^3^ cells/well). After 24 h of incubation at 37°C, the cells were treated with four conditions of TFDG. First, the cells were treated with different concentrations of TFDG (TFDG, 5–30 μM) for 72 h. Second, the cells were treated with different concentrations of pre-treated TFDG (O-TFDG) for 72 h. The concentration of O-TFDG was expressed in TFDG equivalents. Third, the cells were treated by TFDG for only 3 h, after which the media were replaced by fresh media for another 69 h incubation (TFDG-3). Fourth, the cells were treated by O-TFDG for only 3 h, after which the media were replaced by fresh media for another 69 h of incubation (O-TFDG-3). After 72 h, the number of viable cells compared with the control were detected using MTT assay. The treatment media were changed with 100 μl of fresh media containing 0.5 mg/ml of MTT and incubated for 1.5 h. The media were replaced again by 100 μl of DMSO. After gentle shaking, the optical density (OD) was measured at 570 nm using a microplate reader (Elx800 absorbance microplate reader, BioTek Instruments, Inc., Winooski, VT). The cell viability of TFDG-treated cells was normalized to vehicle-treated controls. The inhibition rate was calculated as (1 – OD value of treated cell / OD value of control cell) ×100%.

### Cell-cycle analysis

A cell cycle analysis was performed according to our previous study with slight modifications [17]. Briefly, HCT116 cells were seeded into six-well plates (2 × 10^4^ cells/ml, 2 ml for each well). On the second day, the media were changed, and cells were treated with different concentrations of TFDG and O-TFDG (12.5 and 20 μM). After treatment for 48 h, the cells in the media were collected and fixed in 70% ethanol at −20°C overnight and centrifuged at 4000 rpm for 2 min. Cells were washed twice with ice-cold phosphate-buffered saline (PBS). Samples were stained with propidium iodide (PI, 70 μM) solution containing RNase A (1 mg/ml) in a dark room for 15 min at room temperature, and the cell cycle was analyzed using flow cytometry. For each determination, a minimum of 10,000 cells was counted.

### Apoptosis analysis

The cells were seeded and treated as described in the sextion ‘Cell-cycle analysis’. All cells (both attached and floaters) were harvested on the ice. The harvested cells were stained with Annexin V-FITC in Annexin V staining buffer for 15 min at room temperature and counterstained with PI. Annexin V-FITC measured the translocation of phosphatidylinositol from the inner to the outer cell membrane during early apoptosis, and PI was allowed to enter the cells in late apoptosis or necrosis. Untreated cells were used as a control for the double staining. The cells were analyzed immediately by flow cytometry. For each determination, at least 10,000 cells were analyzed.

### Western blot analysis

Analysis of protein levels was carried out using Western blot. Cell lysates were prepared as described previously [17]. Briefly, HCT116 cells were seeded in 9.6 cm culture dishes (5 × 10^4^ cells/ml, 12 ml for each dish). After 24 h, cells were treated with different treatments of TFDG at the concentrations of 20 and 30 μM. After another 48 h, the media were removed, and cells were washed with ice-cold PBS. Cells were then harvested by adding 50 μl of radioimmunoprecipitation assay buffer containing protease inhibitor 4-(2-aminoethyl) benzenesulfonyl fluoride hydrochloride (AEBSF, 50 mM), phosphatase inhibitor 1 (imidazole sodium fluoride, sodium molybdate, sodium orthovanadate, sodium pyrophosphate tartrate) (1:100), and phosphatase inhibitor 2 (sodium fluoride, sodium orthovanadate, sodium pyrophosphate, β-glycerophosphate) (1:100) (Boston Bioproducts, Ashland, MA). After sonication and centrifugation, supernatants were collected. The protein concentration was detected by the BCA protein assay (Pierce, Rockford, IL). Fifty micrograms of cellular protein was loaded onto 12% sodium dodecyl sulfate-polyacrylamide gels (SDS-PAGE). The separated protein was transferred onto a nitrocellulose membrane (NEN Life Science Products, Boston, MA). After blocking, the membranes were incubated overnight at 4℃ with different primary antibodies in the dark. After incubation with the secondary antibody (Cell Signaling Technology, Inc.) for 2 h, the detection procedure of the proteins bands was carried out with enhanced chemiluminescence measurements (Boston Bioproducts) and according to the manufacturer’s instructions. β-Actin was used as a housekeeping protein. Each experiment was repeated three times.

### Statistical analysis

Experimental values are shown as mean ± SD for triplicate experiments. The statistical significance of the mean difference was analyzed using Student’s two-tailed *t*-test. A one-way analysis of variance (ANOVA) with Bonferroni’s multiple-comparison tests was used when more than two groups’ differences were compared. A significance level of *p* < 0.05 was considered statistically significant.

## Results

### Stability of TFDG in the media

**Figure 2.**
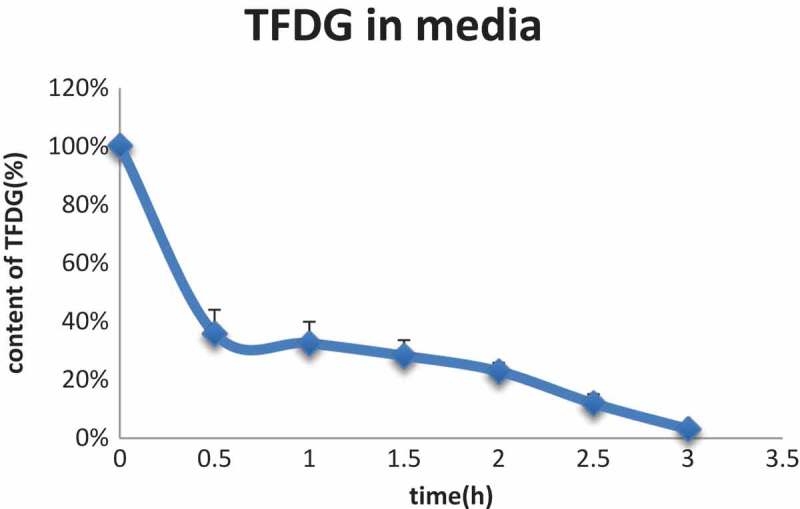
Stability profiles of TFDG (50 μM) in the media at 37°C. Data are presented as means ± SD (*n* = 3).

The factors influencing the stability of polyphenol in cell culture media mainly include the initial concentration of polyphenol, temperature, the presence of metal ions, and the pH value, and pH is possibly the most critical determinant [18]. As with EGCG, TFDG is relatively stable in acidic solutions. However, it is also highly susceptible to degradation in near-neutral or alkaline solution due to the higher number of hydrogen-bond donating hydroxyl groups, leading to further polymerization and decomposition [19]. Theaflavins are more susceptible to degradation than catechins [16]. The stability profiles of TFDG in the media at 37℃ are shown in Figure 2. TFDG decreased rapidly within 3 h.

### Different treatments of TFDG inhibited cell viability of HCT116 cells

**Figure 3.**
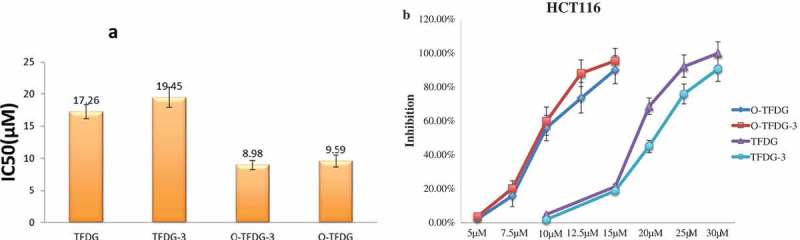
(a) IC_50_ values of different treatments of TFDG on HCT116 cell line. (b) Effect of increasing concentrations of different treatments of TFDG on HCT116 cell proliferation. Values are expressed as means of inhibition rate with respect to control cells ± SD of three replicates.

MTT assays were carried out to examine the effect of different treatments of TFDG on the viability of HCT116 cells. The results showed that different treatments of TFDG inhibited the cell viability of HCT116 cells (Figure 3(b)). The IC_50_ (50% inhibition of cell growth) values were obtained by conducting a conventional WST-1 assay [20]. As shown in Figure 3(b), O-TFDG and O-TFDG-3 significantly inhibited the growth of HCT116 cells when the concentration was >10 μM, which was stronger than TFDG and TFDG-3. The IC_50_ values of TFDG, TFDG-3, O-TFDG, and O-TFDG-3 were 17.26, 19.45, 9.59, and 8.98 μM, respectively (Figure 3(a)). Being consistent with previous reports, TFDG exerts its growth-inhibitory effects against HCT116 cells in a dose- and time-dependent manner. However, no time-dependent inhibitory effect of O-TFDG on HCT116 cells was observed. This could be due to the fact that O-TFDG-3 had a stronger growth inhibition on HCT116 cells than that of O-TFDG, even though this difference did not reach statistical significance.

### Different treatments of TFDG caused cell-cycle arrest in HCT116 cells

**Figure 4.**
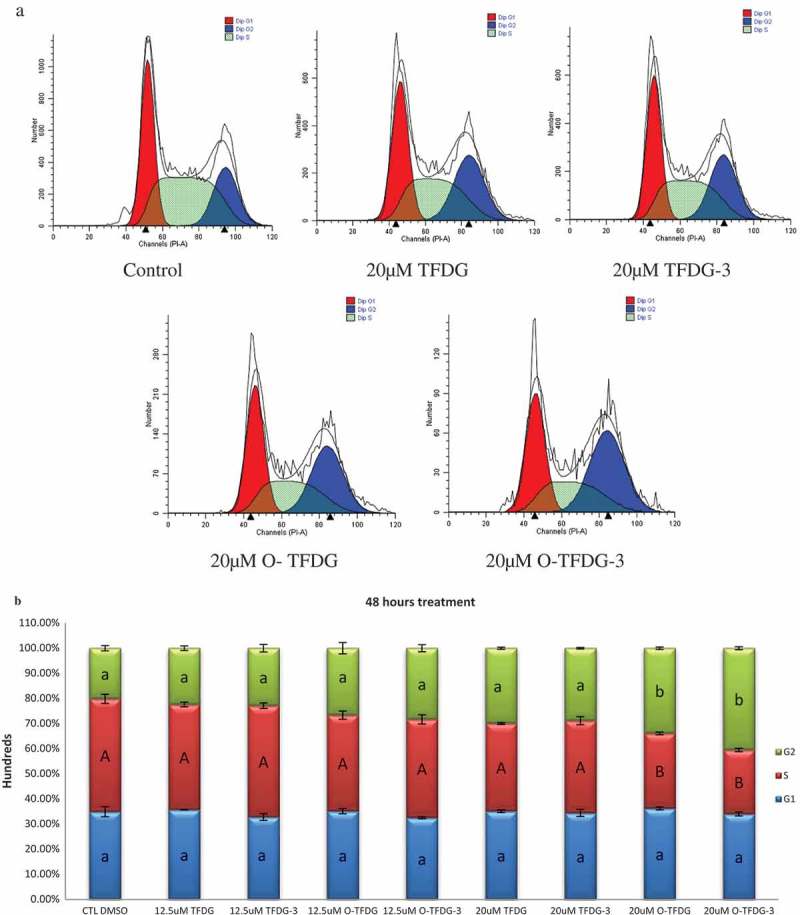
(a) DNA histogram of different treatments of TFDG on HCT116 cells obtained using flow cytometry. (b) Effect of different treatments of TFDG (12.5 and 20 μM) on the HCT116 cell cycle. Comparisions were made using a one-way ANOVA with Tukey’s post hoc test. Different letters (a, A, b, and B) denote significant differnces (*p* < 0.05) between groups, such that groups not sharing a similar letter are significantly different from each other.

Cell-cycle regulation is crucial in the control of cell proliferation and deregulation which cause tumorigenesis [21]. Loss of cell-cycle regulation causes abnormal growth of defective cells, resulting in tumorigenesis. Therefore, cell-cycle blockade is regarded as an effective strategy for eliminating cancer cells. As shown in Figure 4(b), TFDG slightly affected the cell cycle of HCT116 cells when the concentration of TFDG was less than 20 μM, whereas O-TFDG could markedly cause cell-cycle arrest at G2 at the expense of the S phase compared with the control. We also found O-TFDG induced G2 phase cell-cycle arrest in a dose-dependent but not time-dependent manner (Figure 4(b)), because the effect of O-TFDG-3 was better than that of O-TFDG, although the statistical difference is not obvious. Taken together, the inhibitory effect of O-TFDG against proliferation of HCT116 cells was higher than that of TFDG, which might be related to G2 phase cell-cycle arrest.

### O-TFDG increases apoptosis in HCT116 cells

To determine if the loss of viability induced by different treatments of TFDG was due to apoptosis, we detected apoptosis in HCT116 cells using a flow cytometer after Annexin V-FITC/PI staining, which allows quantification of live and dead-apoptotic cells. Concentration dependence for HCT116 cells apoptosis was observed after treatment with O-TFDG for 48 h (*p* < 0.05) (Figure 5(a,b)). Conversely, TFDG with the same dose (i.e. 12.5 and 20 μM) had a slight effect on HCT116 cells, which was consistent with cell-growth inhibition. The apoptotic cells increased from 2.3% to 9.5% when the concentration of O-TFDG-3 increased from 12.5 to 20 μM. Compared with TFDG at the same concentration, the number of apoptotic cells induced by 20 μM of O-TFDG and O-TFDG-3 increased by about four- and eightfold, respectively. Interestingly, there was no time dependence under the same conditions, since 3 h treatment with O-TFDG (O-TFDG-3) was strikingly better than 48 h treatment when the concentration of O-TFDG was 20 μM (the pro-apoptotic effect of O-TFDG treatment on HCT116 cells at 3 h persisted even after a media shift for 45 h). It was also found that O-TFDG induced apoptosis of HCT116 cells mainly appeared within the first 3 hours of treatment, which might be the major pathway for the inhibitory effect of O-TFDG against HCT116 cells.

**Figure 5.**
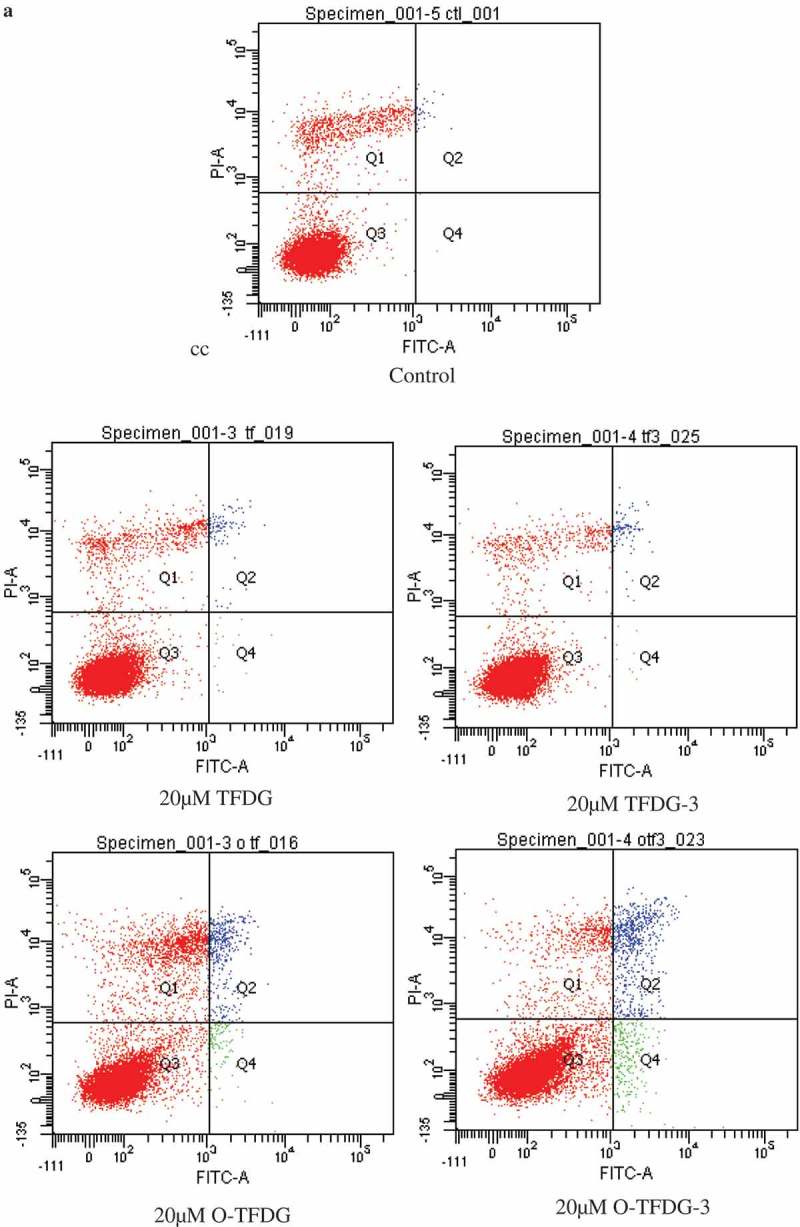
Effects of different treatments of TFDG (TFDG, O-TFDG, TFDG-3, and O-TFDG-3) on HCT116 cells. Cells were treated with different concentrations of different treatments of TFDG for 48 h and then subjected to the annexin V/PI costaining assay. (a) Annexin V/PI costaining dot plots of HCT116 after different treatments of TFDG. (b) Quantification of early and late apoptosis after different treatments of TFDG shown in HCT116 cells.

**Figure 5.**
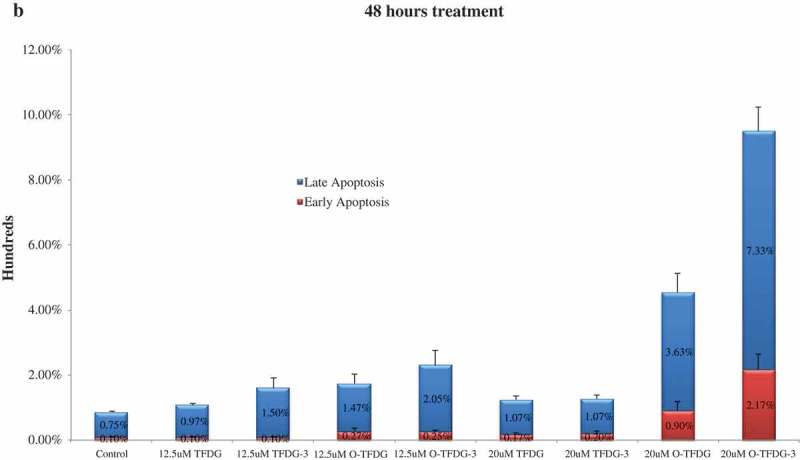
(Continued).

### Effect of different treatments of TFDG on expression of several proteins in HCT116

In order to elucidate the molecular mechanism associated with cell-cycle arrest and apoptosis, cells were treated with different treatments of TFDG (20 and 30 μM) for 48 h, and cell lysates were analyzed by Western blot using antibodies in HCT116 cells. As shown in Figure 6(a,c), p53, p21, cleaved caspase-3, and cyclin D1 were dramatically increased after HCT116 cells were exposed to O-TFDG compared with TFDG. At the same time, the expression of cyclin E was decreased following O-TFDG treatment in a dose-dependent manner. On the other hand, O-TFDG can also inhibit the expression of COX-2, iNOS, and ERK1/2 (Figure6(e)), even though the statistical significance is not obvious due to relatively low concentrations. Interestingly, 3 h treatment with O-TFDG significantly induced overexpression of p21, cleaved caspase-3, and cyclin E in HCT116 compared with both control and 48 h treatment, which was closely associated with O-TFDG-3 induced antiproliferation and apoptosis. This might partially explain why O-TFDG-3 had a better inhibitory effect on HCT116 than that of TFDG and O-TFDG.

**Figure 6.**
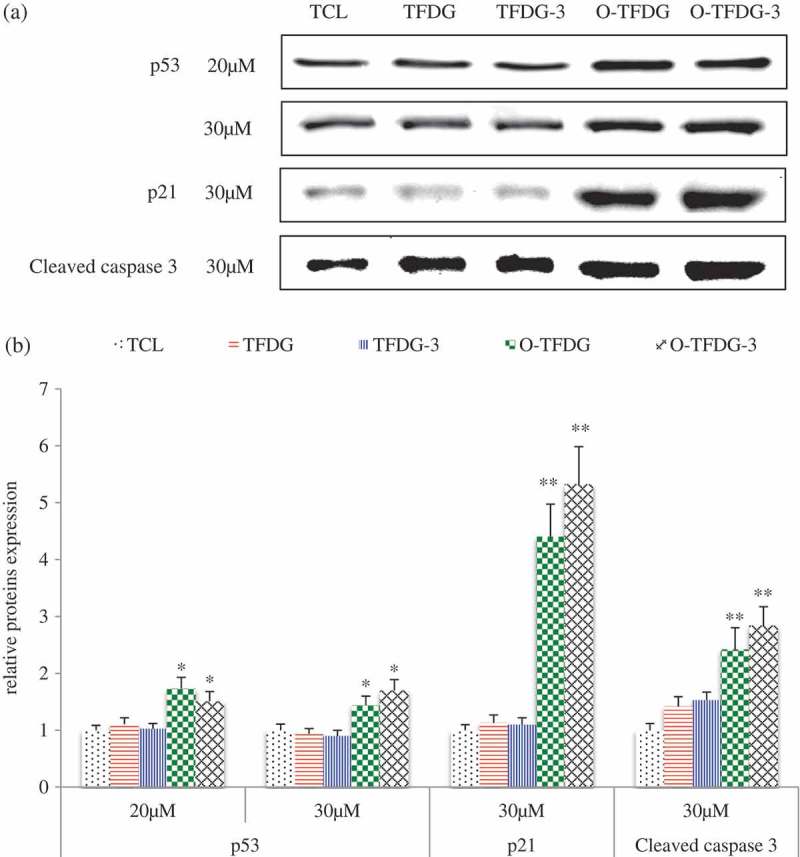
Effect of different treatments of TFDG on P53, P21, cyclin D1, cyclin E, cleaved caspase-3, COX-2, and INOS proteins levels in HCT116 cells. Reproducibility of the data was confirmed by at least three independent experiments. *p < 0.05 and **p < 0.01 compared with the control group.

**Figure 6.**
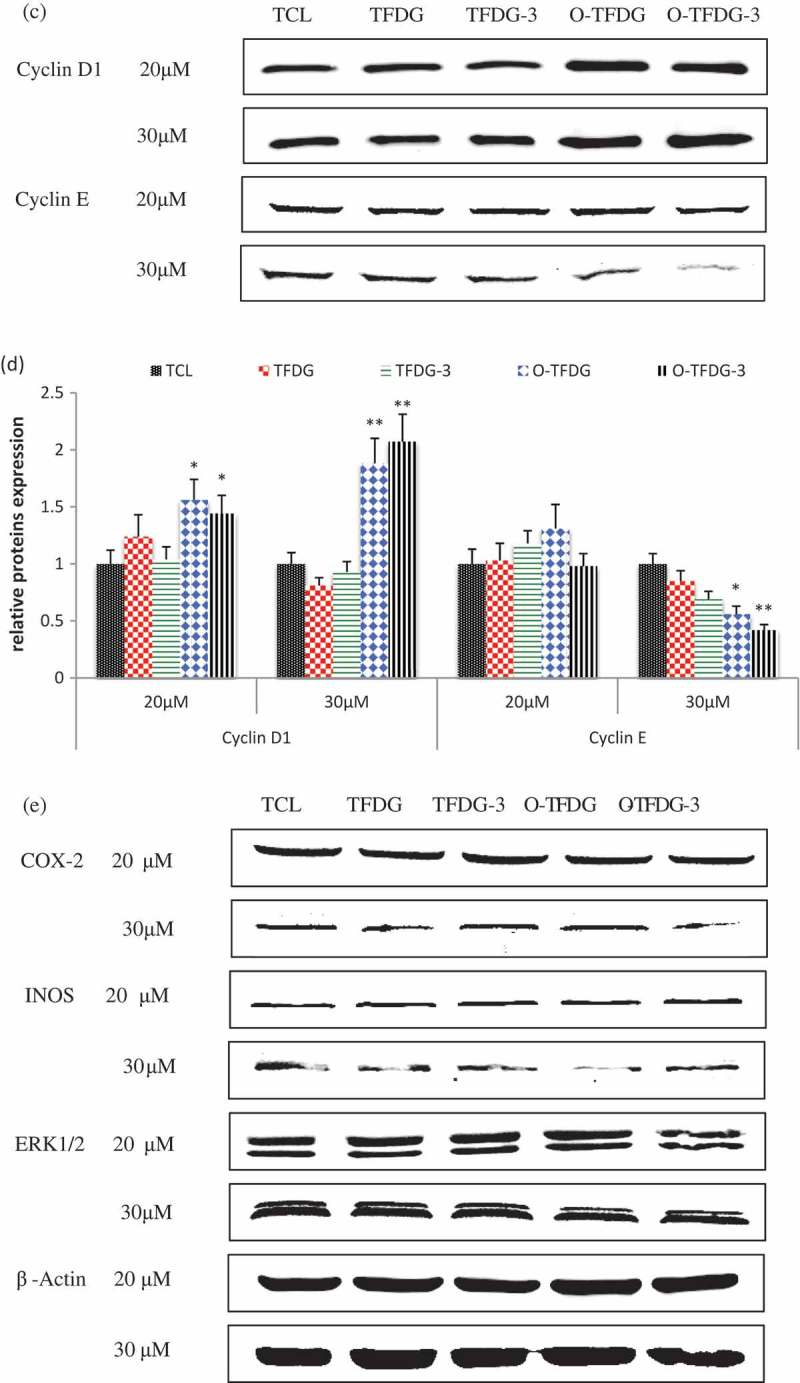
(Continued).

**Figure 6.**
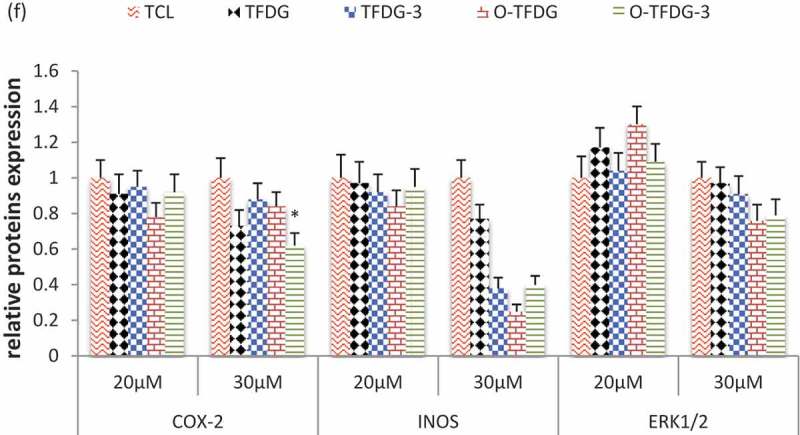
(Continued).

## Discussion

Numerous investigations have indicated that TFDG is a remarkable chemopreventive and chemotherapeutic agent against various cell lines [22,23]. However, TFDG will be quick to completely disappear in physiological conditions, so we speculated that antitumor of TFDG is not completely from TFDG itself. Oxidation products, by-products, and even the intermediates of TFDG might play an important role. Therefore, we investigated the effect of different treatments of TFDG on the HCT116 cell line. In the TFDG-3 groups, TFDG was considered to play a major role in the course of anticancer, whereas in the O-TFDG groups, TFDG was totally ineffective due to the lack of parent TFDG. In this study, we have shown that O-TFDG had a stronger inhibitory effect on HCT116 compared with TFDG, which is consistent with the results from cell cycle and apoptosis analysis. On the other hand, our results revealed that the antineoplastic and pro-apoptotic effect of O-TFDG was not time-dependent, which was different from TFDG, because the increase in the number of dead cells persisted or even more after treated-media removal for the next 21 h.

Chemotherapy-induced inhibitory effect and cell-cycle arrest is usually a prerequisite to the demise of cancer cells. Previous studies showed that the effect of TFs on cell-cycle arrest is still controversial, some reports indicated that TFs can arrest the cell cycle at the G1 phase [24], and others revealed that TFs can induce cell-cycle arrest at the G2 phase [5]. One possible reason is that TFs possess different mechanisms for different cell lines.

The cyclins were named due to the cell-cycle-dependent pattern of expression. They can regulate DNA replication and cell division by activating corresponding cyclin-dependent kinases (CDKs) [25]. Cyclin E can activate CDK2 by the phosphorylation of retinoblastoma protein (Rb), and then promote the release of E2F transcription factors and progression of the cell cycle from the G1 to S phase [26]. Thus, we cannot explain why TFDG can downregulate cyclin E, but induce cell-cycle arrest at G2. Cyclin B1 can cause cell-cycle arrest at the G2 phase, but unfortunately both TFDG and O-TFDG had no effect on this protein (data not shown). p21 with 165 amino acid belongs to the Cip and Kip family of CDK inhibitors, which can directly inhibit the activity of the CDK–cyclin complex. Our data showed that O-TFDG significantly increased p21 levels compared with TFDG, which may be the main reason why O-TFDG had a stronger capacity to cause cell-cycle arrest at G2 than that of TFDG. In addition, Long et al. [27] demonstrated that pyrogallol-type compounds decompose in cell-culture media to generate significant amounts of ROS (such as H_2_O_2_), which initiate signal transduction pathways that are closely related to G2 cell-cycle arrest.

The tumor suppressor protein p53 is known not only to mediate cell-cycle arrest [28] but also to induce cancer cell apoptosis, which is crucial for effective antitumor. It has been shown that p53 triggers apoptosis by inducing mitochondrial outer membrane permeabilization through transcription-dependent and -independent mechanisms [29]. On the other hand, p53 itself may translocate to the mitochondria, directly permeabilizing the outer membrane and causing cytochrome c release by forming complexes with the pro-apoptotic Bcl-2 family proteins and inducing transcription-independent activation of Bax and caspase-8 [30]. Our results revealed that O-TFDG significantly increased p53 levels, but TFDG with the same concentration had little effect on p53, which might be the main reason why O-TFDG had a higher capacity in inducing apoptosis than that of TFDG. Cleaved caspase-3 (C-C-3) is another key mediator of apoptosis. Our studies showed that O-TFDG greatly enhanced the expression of C-C-3 compared with TFDG. However, both had little effect on C-PARP and pro-apoptotic Bax proteins (data not shown). Taken together, it is C-C-3 and p53 pathway by which O-TFDG might induce apoptosis of HCT116 cells. However, the precise mechanism needs to be confirmed by further studies.

Activation of the MAPK extracellular signal-regulated protein kinase (ERK) 1 and 2 generally results in increased cell proliferation and protects cells from apoptosis [31]. Our studies demonstrated that O-TFDG might also induce HCT116 cell apoptosis by decreasing expression of ERK1/2 when the concentration of O-TFDG is >30 μM.

In addition, oxidation reactions and chronic inflammation stimulate cancer cell proliferation and metastasis during the development of tumors [32]. It is now evident that NO plays key roles in regulating the activity of intracellular proteins activating oncogenes, inhibiting tumor suppressor genes to contribute to tumor angiogenesis and invasiveness in human colorectal cancer cells [33], which is produced by inducible nitric oxide synthase (iNOS). Furthermore, NO can also enhance the activity of another proinflammatory enzyme, cyclooxygenase-2 (COX-2) [34], which is upregulated by inflammatory stimuli, such as mitogens, tumor promoters, cytokines, and growth factors [35,36]. Overexpression of protein COX-2 was found in approximately 50% of adenomas and 85% of adenocarcinomas [37] and is associated with worse survival among CRC patients [38], so we speculate that O-TFDG might exert some of their anti-inflammatory and antitumor effects by inhibiting iNOS and COX-2 expression.

In conclusion, the current study demonstrated that O-TFDG had a stronger inhibitory effect on HCT116 cells than TFDG, so we may infer that the degradation products of TFDG play a key role against tumors, especially by-products, such as ROS. It is easy to understand that O-TFDG-3 had a better effect than TFDG-3, because pre-treated TFDG can induce numerous ROS, and the media were replaced with fresh media after only 3 h treatment. However, it is still hard to explain why O-TFDG had a stronger inhibition on HCT116 than parent TFDG, since TFDG have enough time to experience the completely similar process as O-TFDG when they were added into media following 72, 48, or even 24 h treatment. Thus, the only possible explanation is that, for TFDG groups, the cells had a certain time to protect themselves from ROS and/or other degradation metabolites of TFDG to some extent during generation. However, for O-TFDG groups, the cells had no time to respond when numerous ROS and/or other degradation metabolites of TFDG were added immediately. We had found that the same pre-treated EGCG has a similar effect to TFDG on HCT116 cells, so we can speculate that all the polyphenols might share the same mechanism of action on this cell line.

## References

[1] Jemal A, Bray F, Center MM, et al Global cancer statistics. Ca-Cancer J Clin. 2011;61:69–12. 21296855 10.3322/caac.20107

[2] O’Connor ES, Greenblatt DY, Lo Conte NK, et al Adjuvant chemotherapy for stage II colon cancer with poor prognostic features. J Clin Oncol. 2011;29:3381–3388. 21788561 10.1200/JCO.2010.34.3426 PMC3164243

[3] Wang D, Kurasawa E, Yamaguchi Y, et al Analysis of glycosidically bound aroma precursors in tea leaves. 2. Changes in glycoside contents and glycosidase activities in tea leaves during the black tea manufacturing process. J Agr Food Chem. 2001;49:1900–1903. 11308343 10.1021/jf001077+

[4] Bhattacharyya A, Mandal D, Lahiri L, et al Black tea protects imunocytes from tumor induced apoptosis by changing Bcl-2/Bax ratio. Cancer Lett. 2004;209:147–154. 15159016 10.1016/j.canlet.2003.12.025

[5] Prasad S, Kaur J, Roy P, et al Theaflavins induce G2/M arrest by modulating expression of p21^waf/cipl^, cdc25C and cyclin B in human prostate carcinoma PC-3 cells. Life Sci. 2007;81:1323–1331. 17936851 10.1016/j.lfs.2007.07.033

[6] Kalra N, Seth K, Prasad S, et al Theaflavins induced apoptosis of LNCaP cells is mediated through induction of p53, down-regulation of NF-kappa B and mitogen-activated protein kinases pathways. Life Sci. 2007;80:2137–2146. 17499812 10.1016/j.lfs.2007.04.009

[7] Frebourg T. Germline mutations of the p53 gene. Pathol Biol. 1997;45:845–851. 9769948

[8] Scata KA, EI-Deiry WS p53, BRCA1 and breast Cancer chemoresistance. Adv Exp Med Biol. 2007;608:70–86. 17993233 10.1007/978-0-387-74039-3_5

[9] Lahiry L, Saha B, Chakraborty J, et al Theaflavins target Fas/caspase-8 and Akt/pBad pathways to induce apoptosis in p53-mutated human breast cancer cells. Carcinogenesis. 2010;31:259–268. 19969555 10.1093/carcin/bgp240

[10] Adhikary A, Mohanty S, Lahiry L, et al Theaflavins retard human breast cancer cell migration by inhibiting NF-κB via p53-ROS cross-talk. Febs Lett. 2010;584:7–14. 19883646 10.1016/j.febslet.2009.10.081

[11] Alessi DR, Saito Y, Campbell DG, et al Identification of the sites in MAP kinase kinase-1 phosphorylated by p74raf-1. Embo J. 1994;13:1610–1619. 8157000 10.1002/j.1460-2075.1994.tb06424.x PMC394991

[12] Chung JY, Park JO, Phyu H, et al Mechanisms of inhibition of the Ras-MAP kinase signaling pathway in 30.7b Ras 12 cells by tea polyphenols (-)- epigallocatechin-3-gallate and theaflavin-3,3′-digallate. Faseb J. 2001;9:2022–2024. 10.1096/fj.01-0031fje 11511526

[13] Morrison DK, Cutler JRE The complexity of Raf-1 regulation. Curr Opin Cell Biol. 1997;9:174–179. 9069260 10.1016/s0955-0674(97)80060-9

[14] Ross RK, Bernstein L, Lobo RA, et al 5-Alpha-reductase activity and risk of prostate cancer among Japanese and US white and black males. Lancet. 1992;339:887–889. 1348296 10.1016/0140-6736(92)90927-u

[15] Lee HH, Ho CT, Lin JK Theaflavin-3,3′-digallate and penta-O-galloyl- β-D-glucose inhibit rat liver microsomal 5 α-reductase activity and the expression of androgen receptor in LNCaP prostate cancer cells. Carcinogenesis. 2004;25:1109–1118. 14963012 10.1093/carcin/bgh106

[16] Su YL, Leung LK, Huang Y, et al Stability of tea theaflavins and catechins. Food Chem. 2003;83:189–195.

[17] Xiao H, Yang CS, Li S, et al Monodemethylated polymethoxyflavones from sweet orange (Citrus sinensis) peel Inhibit growth of human lung cancer cells by apoptosis. Mo Nutr Food Res. 2009;53:398–406. 10.1002/mnfr.200800057 19065586

[18] Hu J, Chen L, Lei F, et al Investigation of quercetin stability in cell culture medium, role in in vitro experiment. Afr J Pharm Pharmaco. 2012;6:1069−1076.

[19] Xiao JB, HöGger P Stability of dietary polyphenols under the cell culture conditions: avoiding erroneous conclusions. J Agr Food Chem. 2015;63:1547−1557. 25608051 10.1021/jf505514d

[20] Maeda T, Nagaoka Y, Kuwajima H, et al Potent histone deacetylase inhibitors: N-hydroxybenzamides with antitumor activities. Bioorg Med Chem. 2004;12:4351–4360. 15265487 10.1016/j.bmc.2004.06.020

[21] Massague J G1 cell-cycle control and cancer. Nature. 2004;432:298–306. 15549091 10.1038/nature03094

[22] Shao JP, Meng QY, Li YY Theaflavins suppress tumor growth and metastasis via the blockage of the STAT3 pathway in hepatocellular carcinoma. Oncotargets Ther. 2016;9:4265–4275. 10.2147/OTT.S102858 PMC4951064 27478384

[23] Sur S, Pal D, Roy R, et al Tea polyphenols EGCG and TF restrict tongue and liver carcinogenesis simultaneously induced by N-nitrosodiethylamine in mice. Toxicol Appl Pharm. 2016;300:34–46. 10.1016/j.taap.2016.03.016 27058323

[24] Halder B, Gupta SD, Gomes A Black tea polyphenols induce human leukemic cell cycle arrest by inhibiting Akt signaling: possible involvement of Hsp90, Wnt/β-catenin signaling and FOXO1. Febs J. 2012;279:2876–2891. 22715906 10.1111/j.1742-4658.2012.08668.x

[25] Besson A, Dowdy SF, Roberts JM CDK inhibitors: cell cycle regulators and beyond. Dev Cell. 2008;14:159–169. 18267085 10.1016/j.devcel.2008.01.013

[26] Caldon CE, Musgrove EA Distinct and redundant functions of cyclin E1 and cyclin E2 in development and cancer. Cell Div. 2010;5:1–13. 20180967 10.1186/1747-1028-5-2 PMC2835679

[27] Long LH, Hoi A, Halliwell B Instability of, and generation of hydrogen peroxide by, phenolic compounds in cell culture media. Arch Biochem Biophys. 2010;501:162−169. 20558131 10.1016/j.abb.2010.06.012

[28] Basu A, Haldar S The relationship between Bcl2, Bax and p53: consequences for cell cycle progression and cell death. Mol Hum Reprod. 1998;4:1099–1109. 9872359 10.1093/molehr/4.12.1099

[29] Kojima K, Konopleva M, McQueen T, et al Mdm2 inhibitor Nutlin-3a induces p53-mediated apoptosis by transcription-dependent and transcription- independent mechanisms and may overcome Atm-mediated resistance to fludarabine in chronic lymphocytic leukemia. Blood. 2006;108:993–1000. 16543464 10.1182/blood-2005-12-5148 PMC1895860

[30] Chipuk JE, Kuwana T, Bouchier-Hayes L, et al Direct activation of Bax by p53 mediates mitochondrial membrane permeabilization and apoptosis. Science. 2004;303:1010–1014. 14963330 10.1126/science.1092734

[31] Kyriakis JM, Avruch J Mammalian MAPK signal transduction pathways activated by stress and inflammation: a 10-year update. Physiol Rev. 2012;92:689–737. 22535895 10.1152/physrev.00028.2011

[32] Alkhateeb AA, Connor JR The significance of ferritin in cancer: anti-oxidation, inflammation and tumorigenesis. BBA-Rev Cancer. 2013;1836:245–254. 10.1016/j.bbcan.2013.07.002 23891969

[33] Cianchi F, Cortesini C, Fantappie O, et al Inducible nitric oxide synthase expression in human colorectal cancer: correlation with tumor angiogenesis. Am J Pathol. 2003;162:793–801. 12598314 10.1016/S0002-9440(10)63876-X PMC1868089

[34] Nogawa S, Forster C, Zhang F, et al Interaction between inducible nitric oxide synthase and cyclooxygenase-2 after cerebral ischemia. P Natl Acad Sci USA. 1998;95:10966–10971. 10.1073/pnas.95.18.10966 PMC28004 9724813

[35] Minghetti L Cyclooxygenase-2 (COX-2) in inflammatory and degenerative brain diseases. J Neuropath Exp Neur. 2004;63:901–910. 15453089 10.1093/jnen/63.9.901

[36] Simmons DL, Botting RM, Hla T Cyclooxygenase isozymes: the biology of prostaglandin synthesis and inhibition. Pharmacol Rev. 2004;56:387–437. 15317910 10.1124/pr.56.3.3

[37] Gupta RA, DuBois RN Colorectal cancer prevention and treatment by inhibition of cyclooxygenase-2. Nat Rev Cancer. 2001;1:11–21. 11900248 10.1038/35094017

[38] Ogino S, Kirkner GJ, Nosho K, et al Cyclooxygenase-2 expression is an independent predictor of poor prognosis in colon cancer. Clin Cancer Res. 2008;14:8221–8227. 19088039 10.1158/1078-0432.CCR-08-1841 PMC2679582

